# Cervical cell nuclei segmentation based on GC-UNet

**DOI:** 10.1016/j.heliyon.2023.e17647

**Published:** 2023-06-28

**Authors:** Enguang Zhang, Rixin Xie, Yuxin Bian, Jiayan Wang, Pengyi Tao, Heng Zhang, Shenlu Jiang

**Affiliations:** a School of Computer Science and Engineering, Macau University of Science and Technology, Macau, China; b Zhuhai College of Science and Technology, Zhuhai, China; c Faculty of Education, The University of Hong Kong, Pokfulam Road, Hong Kong, China

**Keywords:** Cell nuclei segmentation, Semantic segmentation

## Abstract

Cervical cancer diagnosis hinges significantly on precise nuclei segmentation at early stages, which however, remains largely elusive due to challenges such as overlapping cells and blurred nuclei boundaries. This paper presents a novel deep neural network (DNN), the Global Context UNet (GC-UNet), designed to adeptly handle intricate environments and deliver accurate cell segmentation.

At the core of GC-UNet is DenseNet, which serves as the backbone, encoding cell images and capitalizing on pre-existing knowledge. A unique context-aware pooling module, equipped with a gating model, is integrated for effective encoding of ImageNet pre-trained features, ensuring essential features at different levels are retained. Further, a decoder grounded in a global context attention block is employed to foster global feature interaction and refine the predicted masks.

## 1 Introduction

Cervical cancer, afflicting over 600,000 females and causing roughly 300,000 deaths annually, is one of the most formidable cancers faced by women today. [1], [2]. Early detection can drastically reduce the mortality rate associated with cervical cancer, highlighting the crucial role of swift, accurate diagnosis. The most prevalent diagnostic tools today are Pap smear and liquid-based cytology (LBC), which generate critical imaging data widely used in clinical settings. [3] However, manual screening and analysis of these images present significant challenges: the process is labor-intensive and prone to errors, potentially leading to misdiagnosis. [4], [5].

To address these limitations, research efforts have been channeled towards developing computer-assisted systems capable of automatic image analysis. Such systems typically leverage specific descriptors of abnormal cells in cervical cytology samples and employ machine learning techniques to classify these cells and assist in diagnostic decisions.

Traditional methods for cell segmentation involve a three-step process: cell (cytoplasm and nucleus) segmentation, feature extraction/selection, and cell classification [6], [7], [8]. However, these approaches often struggle with practical application due to their complexity. Poor segmentation results and low accuracy may occur due to background noises and overlapping cell clusters. More recent approaches have streamlined the process using deep neural networks (DNNs) for semantic segmentation, which requires just one step: input the image and output the label map for cells [9], [10], [11].

While public datasets for cervical cytology exist, enabling model training, most research has focused on segmentation of overlapping cytoplasm and cell nuclei [12], [13], [14]. The complexities of real-world clinical scenarios, such as frequent overlaps or deformities in nuclei smears or similar colors between nuclei and overlapped cytoplasm, are often not fully reflected in these datasets. [15], [16], [17], [18]. Consequently, current methods often fall short when applied to cervical cell images in real-world settings.

The key contributions of this paper include:

1. The development of a context-aware gating pooling system that extracts features from multiple scales. Our multi-scale context gating module aids spatial feature extraction, while our global context attention module helps establish texture dependency across scales.
2. The introduction of a Residual Context Attention decoder that captures scale dependency from multi-scale features, resulting in precise prediction masks.
3. Demonstrable superior accuracy of the proposed method compared to state-of-the-art approaches across five typical medical image processing datasets. Additionally, results from real-world evaluations indicate the potential of our method in detecting cervical nuclei at early stages.

## 2 Related work

Cell nucleus segmentation has been extensively studied due to its crucial role in assisting with medical diagnoses, particularly in the realm of cervical cell nuclei. This section will review existing methods and highlight the distinguishing characteristics and advancements of our proposed approach. Numerous methods for cell nucleus segmentation have been proposed over the years.

Early approaches used classical feature methods, e.g., canny detection [19], watershed method [20], feature cluster [21], binary threshold [22], [23], image enhancement [24] etc. However, these hand-crafted features often fell short when dealing with complex background noise and environmental variations, resulting in poor quality segmentation.

Attempts were made to mitigate these shortcomings by incorporating machine learning techniques. For instance, some researchers used a mean-shift classification method [25], which uses dynamic threshold to segment cell images. But these results proved sensitive to abnormal environments and, in most cases, traditional features struggled to yield promising outcomes.

The advent of deep neural networks has revolutionized cervical cell nucleus segmentation, providing significant improvements over traditional methods [20], [21], [22]. For example, Mask-RCNN was used for instance segmentation [26], and local fully connected conditional random fields (LFCCRF) were employed to refine the clear boundary of nuclei [15]. Additionally, multiple classification methods were deployed to classify both cytoplasm and nucleus at the pixel level based on super-resolution methods. Some researchers even used their proprietary datasets to enhance training procedures [18]. The CNN Bi-path architecture was utilized to segment Pap smear images and classify cervical cancer, achieving substantial improvements in accuracy [27].

Deep Neural Networks (DNNs) like U-Net, CENet, TripleUNet, and HoverNet have been used for nucleus detection in multi-organ segmentation datasets (MoNuSeg, CoNSeP, and CPM-17). U-Net [28], particularly, has gained popularity in medical image segmentation, with several extensions being proposed. For instance, the CENet method employed DAC and RMP Blocks ased on UNet for medical image segmentation [29]. TripleUNet incorporated a hematoxylin-aware module [30]. Techniques like CIA-Net predicted clear nuclei boundaries through a contour-aware module, while Hover-Net [13] used pixel-to-centroid distance maps. The accuracy of these approaches often heavily relies on post-processing.

Distinct from previous studies, our proposed GCUNet presents a unique integration of well-established components including the DenseNet backbone, the Context Gating Pooling (CAGP) block, the Context Gating Residual (CGR) block, and the Residual Context Attention (RCA) module. While these modules have been explored individually in existing literature, our work distinguishes itself by amalgamating these components in a novel way that enhances feature extraction and encoding, preserves spatial resolution, and endows the model with a level of context-awareness. In addition to this, our approach to employ Multi-path Residual Pooling for efficient multi-scale dense feature extraction offers a distinctive edge to our work. The strength and novelty of our research stem not just from the individual components it incorporates, but primarily from their unique assembly and application.

## 3 Research methodology

In this part of the paper, we detail the GC-UNet architecture's inner workings and structure.

### 3.1 Overall architecture

**Figure 1:**
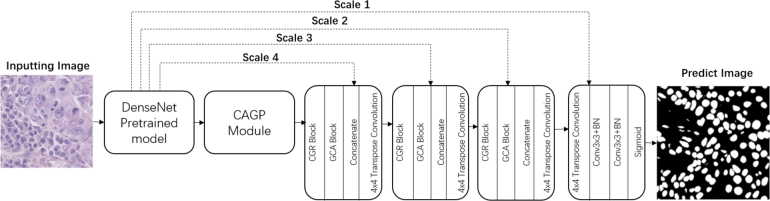
Overall architecture of proposed GCUNet.

We created the GCUNet, basing its structure on fundamental deep neural networks, namely UNet and CENet. As depicted in Fig. 1, the encoder block in our design uses the DenseNet Block [31] which is pretrained using ImageNet [32], which significantly differs from the original U-Net. Unlike U-Net, which features skip connections between corresponding encoder and decoder layers, DenseNet presents a dense connectivity pattern where each layer connects to every other layer in a feed-forward fashion. This facilitates feature reuse, mitigates the vanishing gradient problem, and reduces parameters, making training more efficient. This efficient and detailed extraction and propagation of features from various scales is the primary advantage of using a DenseNet block, thus improving nuclei segmentation performance in GCUNet. Our Context-Aware Gate Pooling module (CAGP) facilitates the extraction of more high-level semantic feature maps. Additionally, we present two innovative decoders in this work, the Residual Context Attention Decoder (GCAD) and the Global Context Attention Decoder (GCA). These decoders serve the purpose of predicting the label map.

### 3.2 CAGP module

**Figure 2:**
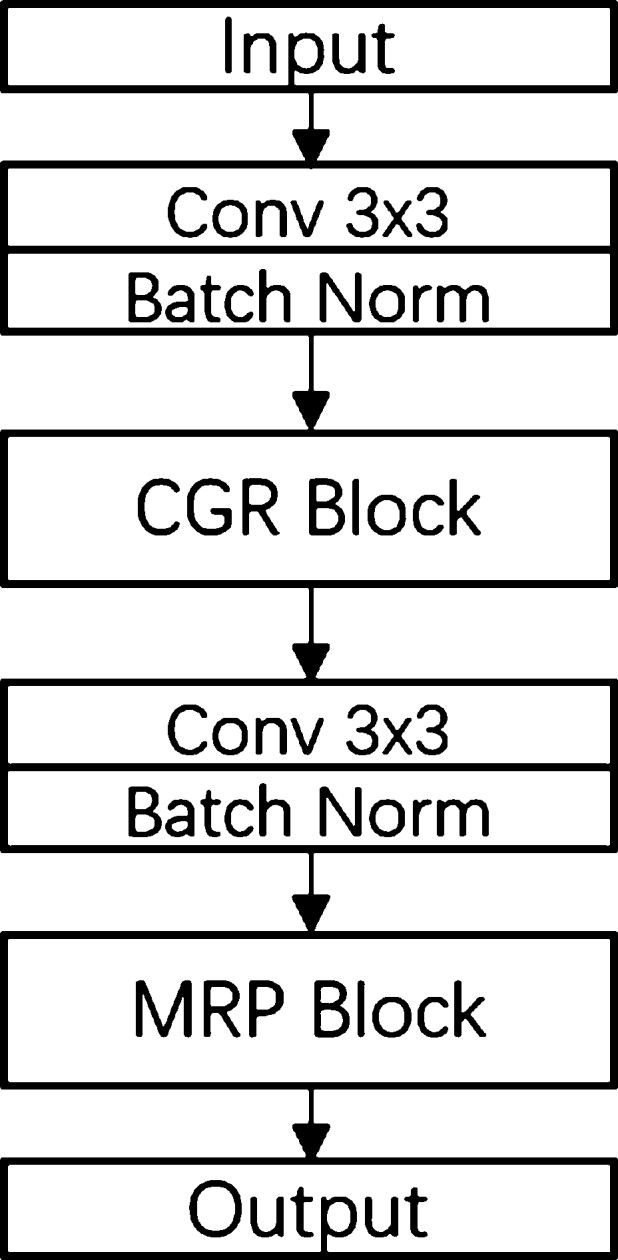
The architecture of CAPG module.

The CAGP module, visualized in Fig. 2, is designed to extract features from multiple scales effectively and retain them for decoder utilization.

#### 3.2.1 Context gating residual (CGR) block

**Figure 3:**
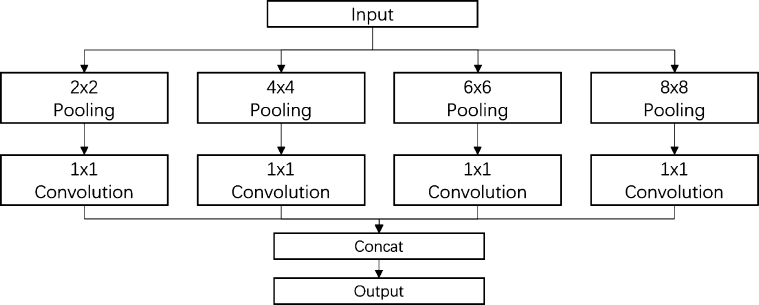
The architecture of CGR block.

The GC module [33] is a highly efficient non-linear component designed to capture the intricate relationships among network activations. It is visualized in Fig. 3. The formula is defined in equation (1):

(1) $$I^{\prime }=F\cdot \;\sigma \left(W⁎I+a\right)$$

where $I\in \;R^{n}$ the input, *σ* reveals sigmoid function [34], and ⨀ means multiplication. $I\in \;R^{n\;\times \;n}$ and $a\;\in \;R^{n}$ mean the trainable parameters. The weights σ(W⁎I+a)∈(0,1] is a series of trained gates for individual scale of the *I*.

The Context Gating Residual (CGR) Block, depicted on Fig. 3, to increase the receptive field in the context gating module. It generates four paths based on two convolution layers with varying kernel sizes (3, 5, 7, and 9) and a residual skipping connection. These paths generate attention weights on a specific scale which are multiplied with feature maps to calculate weighted feature maps from all scales. The CGR Block then amalgamates all these feature maps with the input from the residual skipping connection branch to form a final feature map based on multi-scale information. This design enables the avoidance of redundant features and maximizes the use of original input cell images. This component is key to enhancing feature maps by leveraging contextual information across different scales and directions. The CGR Block's capability to capture both local and global spatial information via a residual connection effectively expands the receptive field. As a result, the CGR Block provides a powerful mechanism for extracting critical features from the image's context. This aspect significantly improves the context gating module's efficacy, which in turn contributes to the superior performance of the GCUNet in nuclei segmentation tasks.

#### 3.2.2 Global context attention (GCA) block

**Figure 4:**
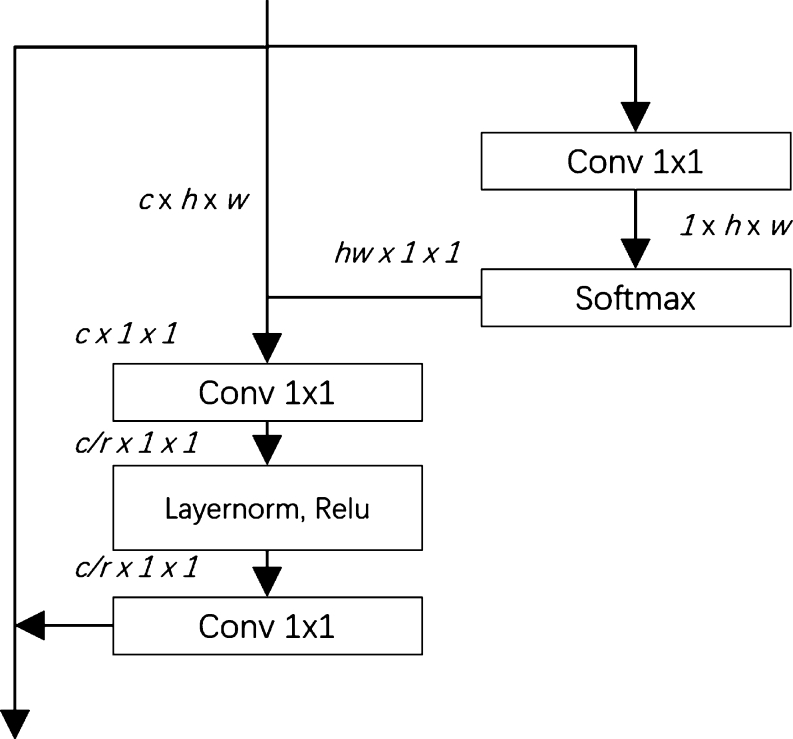
The architecture of GCA Block.

The utilization of context information to facilitate better cell map segmentation has been demonstrated in various research studies [35]. As a result, we introduce the GCA Block [36], after the convolution process, the multi-scale fusion information is utilized to enhance the receptive field and adjust the weighting of each scale. This structural adjustment allows the network to filter trivial elements effectively and maintain the high performance of the proposed network. The GCA Block architecture is shown in Fig. 4.

The inputting feature map is defined as $I^{\prime }\in R^{C\;\times \;h\;\times \;w}$, the procedure of calculation is defined as follows:

1.) The left path utilizes 1×1 convolution to $I^{\prime }$ to obtain a modified feature map in size (1,h,w]. Then it is reshaped to (hw,1,1] for further processing. Meanwhile, the softmax is applied on the reshaped feature map. Then the second path modifies the shape of $I^{\prime }$ to (c,hw]. Finally, the two paths are multiple to get the new feature map $I_{t}\in R^{C\;\times \;1\;\times \;1}$. And the function can be defined in equation (2):
  (2) $$I_{t}=R_{r}(I^{\prime })⊗\;s\left(R_{r}(C(I^{\prime }))\right)$$
  Where the C(•) is convolution operation, s(•) is softmax operation, $R_{r}$ is reshape operation, and ⊗ denotes matrix multiplication.
2.) To minimize parameters after the 1×1 convolution, feature $I_{t}$ is reshaped to size $\left(\frac{c}{r},1\;,\;1\right]$, and *r* is the ratio of bottleneck which is set to 16. The layer normalization (LN) and the ReLU activation function are employed to enhance the feature generalization capability on DN. Finally, the feature map is resized to original resolution through equation (3):
  (3) $$I^{\prime }=I⊕\;C\left(s_{ln+ReLu}C(I_{t})\right)$$
  Where the ⊕ denotes the channel-wise summation operation, and $s_{ln+ReLu}$ means LN followed by ReLu.

In our GCUNet, we incorporate a critical component, the Global Context Attention block. This element is key to recalibrating feature responses in light of their global contextual significance. Through consideration of the global context, the GCA block is able to discern and prioritize the most pertinent features for a given task, which effectively expands the receptive field of our network. Furthermore, the GCA block applies a re-weighting strategy to accentuate crucial features while simultaneously diminishing less significant ones. This is achieved by generating attention maps for each scale. These maps are subsequently utilized to modulate the feature responses, enabling the network to focus more intently on relevant features. The inclusion of the GCA block thus plays a crucial role in the superior performance of our proposed GCUNet in nuclei segmentation tasks.

#### 3.2.3 Multipath residual pooling (MRP) module

**Figure 5:**
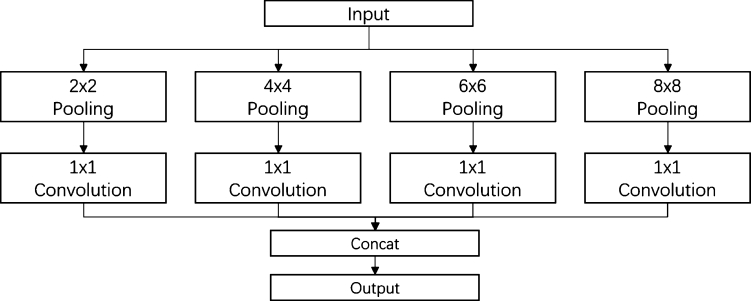
The architecture of MRP module.

The MRP module, shown in Fig. 5, enhances the traditional max pooling operation by switching from a single kernel size to a larger receptive field. This switch allows the neural network to form stronger internal connections, leading to a richer context feature description for subsequent decoding. In this work, we use four max pooling paths (2x2, 3x3, 5x5, and 7x7) and a 1x1 convolution filter to adjust the pooled feature maps and make them more adaptive. The four paths are then integrated using a concatenation operation as output. This process creates a larger receptive field for classifying cells and background, helping to avoid the overlapping issues prevalent in smear images.

### 3.3 Feature decoder module

**Figure 6:**
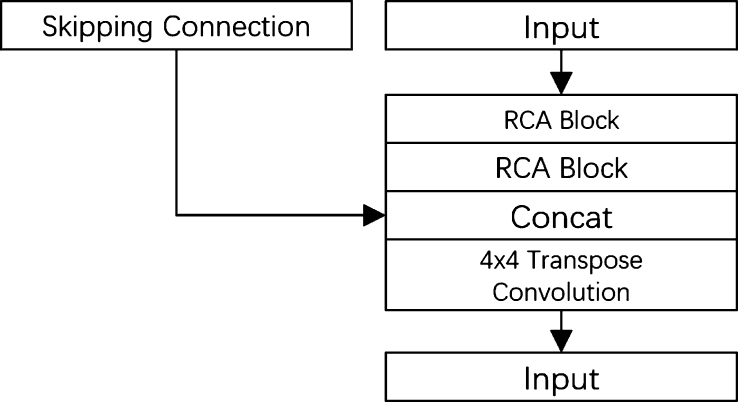
Architecture of feature decoder module.

Given that the image size is inevitably reduced during feature extraction, we employ four decoder blocks, shown in Fig. 6, to restore the size to the original image dimensions. The Feature Decoder Module in GCUNet applies a 4x4 transposed convolution to upscale the feature maps, two 3x3 convolutions to extract local features, and Batch Normalization for feature standardization to improve learning stability and speed. A sigmoid activation function is used to convert the network's output into pixel-wise class probabilities, crucial for segmentation tasks. The decoder produces a mask of the same size as the original input, and skipping connections are employed to transfer original features from each encoder to each decoder.

**Figure 7:**
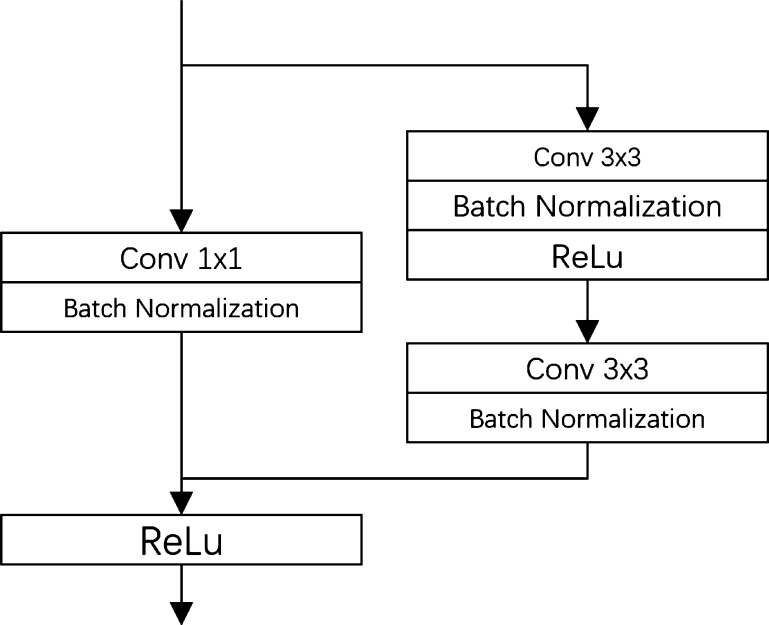
Architecture of RCA module.

Inner the decoder the Residual context attention module (RCIA) is also employed. As shown on Fig. 7, the Residual Context Attention (RCA) module within the decoder generates two paths: a global context path and a local feature path. The global context path utilizes a pyramid pooling structure to extract global information across the image, while the local feature path is designed to maintain the original input features for more detailed, context-specific information. The combination of these two paths enables the model to handle complex segmentation tasks effectively by leveraging both local and global feature information.

## 4 Experiments and discussion

This section presents our experimental approach, designed to validate the effectiveness of our proposed GC-UNet. We explain the evaluation metrics, datasets, experimental setup, and offer quantitative evaluations for consideration.

### 4.1 Evaluation metrics

To benchmark our proposed GC-UNet against contemporary deep learning methodologies, we have employed widely used evaluation metrics for this task, namely, aggregated jaccard index (equation (4)), dice coefficient (equation (5)), and panoptic quality.

(4) $$AJI=\frac{\sum_{i=1}^{n}G_{i}\cap P_{j}}{\sum_{i=1}^{n}G_{i}\cup P_{j}+\sum_{k\in N}P_{k}}$$

Where $j=argmax\;(\frac{G_{i}\cap \;P_{k}}{G_{i}\cup P_{k}})$, where $G=\left(G_{1},\;G_{2},\;\ldots G_{n}\right\}$, $P=\left(P_{1},\;P_{2},\;...P_{m}\right\}$ denote the ground truth and the prediction results. And, *N* denotes a set of indices of prediction results without any corresponding ground truth. Dice coefficient measures the overlapping degree between the two regions and is given by

(5) $$Dice=\;\frac{2\;\times \;|G\;\cap P|}{\left|G|+|P\right|}$$

Given that the aforementioned two methods are not sensitive to overlapping regions with false-positive results, we also implement the Panoptic Quality (PQ) to evaluate the nuclei segmentation. PQ evaluates the prediction segment and the ground truth at the instance level, employing the Intersection over Union (IoU) method.

A (p, g) pair is considered correct if the IoU exceeds 0.5. True Positives (TP) refer to matched pairs of segments, False Positives (FP) indicate unmatched predicted segments, and False Negatives (FN) stand for unmatched ground truth segments, respectively.

We selected the Aggregated Jaccard Index (AJI), Dice Coefficient, and Panoptic Quality (PQ) as our evaluation metrics due to their unique capabilities in assessing model performance. AJI provides a robust measure of segmentation accuracy, accounting for both intersection and union of the predicted and ground truth segments. The Dice Coefficient, on the other hand, measures similarity between the predicted and actual segments, effectively indicating precision and recall. PQ captures both segmentation and detection tasks in a single score, offering a more comprehensive evaluation. It is important to note, however, that each of these metrics has its own strengths and limitations. While AJI is robust, it can be overly sensitive to slight inaccuracies in segment boundaries. The Dice Coefficient, though effective in measuring overlap, doesn't account for false negatives and false positives separately.

### 4.2 Dataset and pre-processing

To validate the efficacy of our proposed method, we employed five public datasets for a quantitative evaluation. The first one is the MoNuSeg Dataset [12], consisting of 30 images of size 1000x1000, cropped from whole slide images. We chose 16 for training and 14 for testing. The second is the CoNSep dataset [13], comprising 41 H&E stained images of size 1000x1000 at 40x magnification, extracted from 16 CRA WSIs. Here, we chose 27 for training and 14 for testing. Finally, CPM-17 [14] is used as the third dataset, containing 40 pathological images from which we chose 32 for training and 8 for testing. Each image is scanned at 40x magnification and measures 500x500 pixels. The fourth dataset is the TCGA-KUMAR database [37], which includes 30 labeled images of size 1000×1000 at 40× magnification from The Cancer Genome Atlas (TCGA). The images come from seven different organs: breast, bladder, colon, kidney, liver, prostate, and stomach, with 15 images selected for training and 15 for testing. The final one is TNBC dataset [38], containing 50 annotated images of size 500×500 collected from 11 different patients of the Curie Institute. The dataset was split into 30 training images and 20 test images. A brief summary of these datasets and their attributes is shown in Table 1.

**Table 1:** Brief summary of these datasets and their attributes.

Dataset	# of Images	Image Size	Division
MoNuSeg	30	1000x1000	16/14
CoNSeP	41	1000x1000	27/14
CPM-17	40	500x500	32/8
TCGA	30	1000x1000	15/15
TNBC	50	500x500	30/20

The image preprocessing steps were vital in our work to ensure consistent input to the model and maximize the learning efficacy. The preprocessing techniques we applied include:

**Image Resizing:** All images were resized to a uniform size to maintain consistency in the model input.
**Image Rotation:** To augment the dataset and improve the model's robustness, images were rotated at various angles.
**Image Translation:** Images were translated horizontally and vertically to create more diverse training examples.
**Data Normalization:** Pixel intensities were scaled between 0 and 1 to help the model converge faster during training.

**Figure 8:**
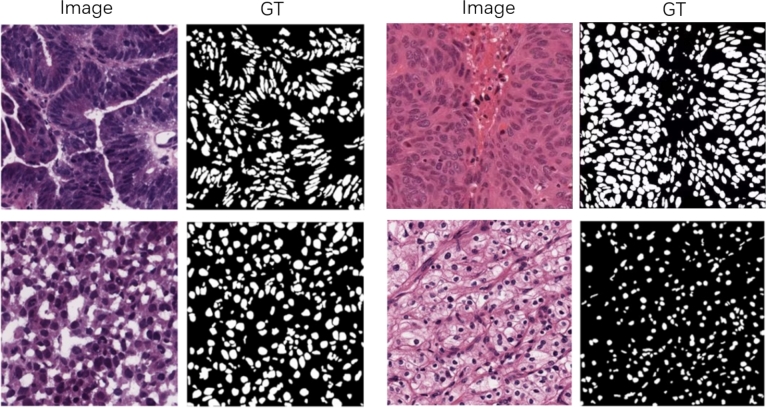
Sample of dataset. Image means the original image. GT means the ground truth labels corresponding to images.

The above methods for data argumentation is processed in each epoch, and enlarge the amount of training dataset to its 10 times comparing with original size. The sample of the dataset for training is shown on Fig. 8.

### 4.3 Implementation detail

The GC-UNet model utilizes a Ryzen 5800 CPU, 64 GB DDR4 RAM, and a Titan RTX (24 GB) GPU for training and testing. The model is trained over 200 epochs, taking a total of 1.2 hours, using the Adam optimizer with a starting learning rate of 0.0001 and a batch size of 8. The loss function employs a combination of binary cross-entropy and dice loss. All images are resized to 512x512 for both training and testing purposes. Additionally, common data augmentations are applied to this task [36].

### 4.4 Training procedure

**Figure 9:**
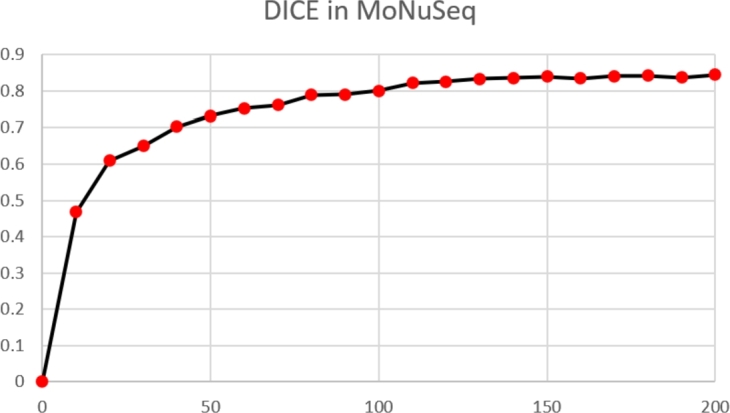
Accuracy vs Training Epoch.

**Figure 10:**
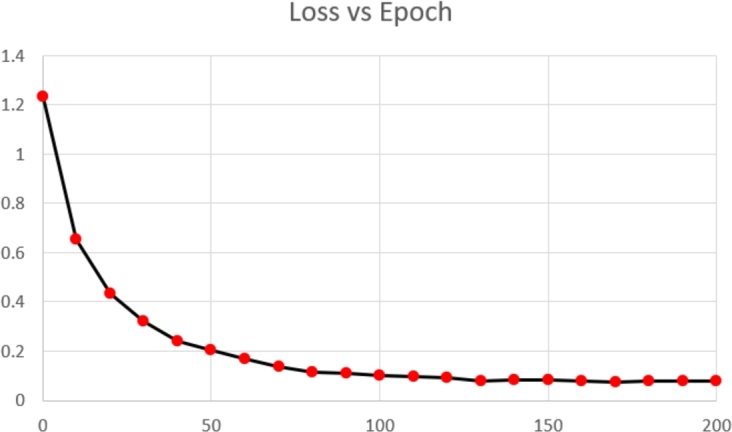
Loss vs Training Epoch.

Figure 9, Figure 10 showcases the evolution of the model's performance during training, highlighting the consistent improvement in accuracy and decrease in loss over epochs. Starting at epoch 0, the model registered a high initial loss of 1.232. However, as the training advanced, the model learned effectively from the data, reducing the loss to 0.652 at the 10th epoch and further to 0.434 by the 20th epoch.

The downward trend in loss continued steadily, with the loss falling to 0.321 at the 30th epoch, and the model achieved a loss of 0.205 by the 50th epoch. This significant reduction in loss denotes the model's successful learning. As the epochs progressed, the model continued to minimize the loss, achieving a loss of 0.102 by the 100th epoch. At the final 200th epoch, the loss had been further reduced to 0.078.

Simultaneously, the model's accuracy consistently increased from 0 at the initial epoch to 0.845 by the 200th epoch, signifying the model's ability to better predict the nuclei segmentation in biomedical images.

The simultaneous decrease in loss and increase in accuracy illustrates the effectiveness of the GCUNet model and its robust learning capability. However, we observe minor fluctuations in loss and accuracy in later epochs, indicating the model's oscillation around an optimal solution as the training converged. This balance between the loss decrease and accuracy increase over time substantiates the chosen model parameters and training strategy.

### 4.5 Ablation study

We conducted an ablation study, comparing the original UNet model with/without each module across the five public datasets. The results are represented in Table 2. The configurations tested are as follows:

1.) UNet: the original basic network.
2.) ResNet: UNet's encoder is replaced by ResNet 101 [39]. (R)
3.) DenseNet: UNet's encoder is replaced by DenseNet [31]. (D)
4.) MobileNet: UNet's encoder is replaced by MobileNet [40], (M)
5.) DenseNet + CAGP Block: UNet is replaced by DenseNet with a CGR block. (D+CAGP)
6.) DenseNet + CAGP Block + CGR module: UNet is replaced by DenseNet, with a CGR block and CG Residual (CGR) Block. (D+CAGP+CGR)
7.) DenseNet + CAGP Block + CGR module + Multipath Residual Pooling (MRP) Module: UNet is replaced by DenseNet, with a CGR block, CG Residual (CGR) Block and Multipath Residual Pooling (MRP). (D+CAGP+CGR+MRP)
8.) DenseNet + CAGP Block + CGR module + Multipath Residual Pooling (MRP) Module + Residual context attention (RCA) module: UNet is replaced by DenseNet, with a CAGP block, CG Residual (CGR) Block, Multipath Residual Pooling (MRP), and Residual context attention (RCA) module. (D+CAGP+CGR+MRP+RCA).

**Table 2:** Ablation study of GCUNet with/without different components.

Model	MoNuSeg			CoNSeP			CPM-17		
	AJI	DICE	PQ	AJI	DICE	PQ	AJI	DICE	PQ
UNet	0.515	0.775	0.490	0.479	0.740	0.405	0.554	0.810	0.501
R	0.554	0.783	0.518	0.550	0.752	0.447	0.615	0.847	0.550
D	0.590	0.802	0.534	0.568	0.817	0.535	0.654	0.867	0.594
M	0.503	0.712	0.447	0.453	0.694	0.369	0.512	0.781	0.475
Item 4	0.567	0.813	0.561	0.578	0.810	0.552	0.683	0.878	0.612
Item 5	0.602	0.825	0.589	0.540	0.823	0.579	0.694	0.882	0.625
Item 6	0.638	0.841	0.625	0.621	0.834	0.575	0.712	0.890	0.623
Item 7 (SA)	0.624	0.829	0.605	0.630	0.819	0.572	0.705	0.875	0.618
Item 7 (CBAM)	0.630	0.833	0.609	0.627	0.825	0.581	0.720	0.891	0.644
Item 7 (SE)	0.641	0.841	0.620	0.625	0.833	0.575	0.722	0.901	0.632
Item 7	0.648	0.845	0.630	0.629	0.836	0.580	0.724	0.895	0.651

In addition, we also tested the Shuffle Attention (SA) [41], CBAM attention, [42] and SE attention [43] instead of global context attention in our ablation study.

Table 2 reveals that leveraging pre-trained backbones significantly enhances performance across all three datasets. Considering the MoNuSeg dataset, for instance, the ResNet model achieves 0.554 in AJI, 0.783 in DICE, and 0.518 in PQ. These results are respectively about 0.39, 0.08, and 0.28 higher than those of the basic UNet. DenseNet, with its superior deep feature encoding ability, performs even better, yielding 0.590, 0.802, and 0.534 in AJI, DICE, and PQ respectively.

The superior performance of DenseNet is attributed to its ability to adapt features for encoding cells, given that ImageNet, which forms its training base, does not contain medical images. This enables it to outperform both ResNet and MobileNet, which are not as efficient in connecting multi-scale feature information. Conversely, MobileNet, being a lightweight backbone, performs slightly poorer than the basic UNet, achieving 0.503, 0.712, and 0.447 in AJI, DICE, and PQ respectively.

Similar results are observed for CoNSeP and CPM-17 datasets, with DenseNet yielding the highest scores - 0.568, 0.817, and 0.535 in CoNSeP, and 0.654, 0.867, and 0.594 in CPM-17, respectively. Consequently, due to DenseNet's superior encoding ability observed from the ablation study, it is selected for subsequent evaluations.

Next, we compared the results of D+CAGP, D+CAGP+CGR, D+CAGP+CGR+MRP, and D+CAGP+CGR+MRP+RCA configurations. Our findings reveal that all configurations perform better than those without enhancements, and our proposed method (D+CAGP+CGR+MRP+RCA) yields the highest results: 0.648, 0.845, 0.630 in MoNuSeg; 0.629, 0.836, 0.580 in CoNSeP; and 0.724, 0.895, 0.651 in CPM-17.

Aside from evaluations employing global context attention, we also tested the Shuffle Attention (SA), CBAM attention, and SE attention. Global context attention consistently achieved the best results in MoNuSeg and CPM-17, advancing by about 1.5% in all evaluation metrics and maintaining similar accuracy levels in CoNSeP. This outcome stems from global context attention's large receptive field during decoding, which aids the encoder in extracting cell boundaries at multiple scales. Hence, we determined that global context attention is most suitable for the task of nuclei segmentation and will be employed in further studies.

The data in Table 1 demonstrate that each component influences the performance of nuclei segmentation and that our proposed GC-UNet model consistently outperforms other configurations. In subsequent evaluations, we will directly compare the performance of GC-UNet with other models.

### 4.6 Quantitative comparison

In this section, we examine the performance of seven state-of-the-art methods across five datasets: UNet, UNet++, Attention UNet, CENet, TripleUNet, GCPNet, and HoverNet. Each model was trained multiple times, with the best-performing instance represented in the table. The average mean and standard deviations for the five datasets are as follows: UNet (0.0152 and 0.00023), UNet++ (0.03114 and 0.00097), CENet (0.03674 and 0.00135), TripleUNet (0.01643 and 0.00027), HoverNet (0.01517 and 0.00023), GCPNet (0.0189 and 0.00036), and GCUNet (0.017 and 0.00029).

As illustrated in Table 3, the proposed CGUNet maintains a high degree of accuracy in comparison to other state-of-the-art methods. Methods that utilized an attention module and transfer learning (UNet++, Attention UNet, CENet, TripleUNet, HoverNet, GCPNet) consistently outperformed the original UNet. Among these, GCPNet demonstrated impressive results across all datasets. This superior performance is attributed to the attention module's capacity to effectively extract features and generate context feature information, a clear advantage over the unoptimized original UNet.

**Table 3:** Quantitative evaluation comparing GCUNet with other state-of-art methods.

Model	MoNuSeg			CoNSep			CPM-17			TCGA			TNBC		
model/ merit	AJI	DICE	PQ	AJI	DICE	PQ	AJI	DICE	PQ	AJI	DICE	PQ	AJI	DICE	PQ
UNet	0.515	0.775	0.490	0.479	0.740	0.405	0.554	0.810	0.501	0.528	0.789	0.575	0.564	0.752	0.570
UNet ++	0.612	0.805	0.565	0.558	0.824	0.532	0.640	0.858	0.601	0.586	0.795	0.591	0.581	0.776	0.605
Att-UNet	0.582	0.796	0.521	0.535	0.811	0.528	0.634	0.838	0.592	0.538	0.756	0.550	0.578	0.765	0.593
CENet	0.530	0.792	0.504	0.492	0.765	0.426	0.645	0.868	0.615	0.531	0.790	0.572	0.569	0.802	0.615
Triple UNet	0.621	0.835	0.605	0.585	0.842	0.571	0.702	0.862	0.643	0.601	0.810	0.610	0.615	0.795	0.620
Hover Net	0.628	0.826	0.602	0.579	0.844	0.532	0.692	0.852	0.653	0.611	0.821	0.630	0.631	0.812	0.621
GCP Net	0.632	0.834	0.623	0.601	0.840	0.575	0.715	0.869	0.650	0.610	0.825	0.627	0.654	0.804	0.630
GCU Net	0.648	0.845	0.630	0.629	0.836	0.580	0.724	0.895	0.651	0.625	0.835	0.638	0.657	0.810	0.634

However, the GCUNet consistently outperformed all other models, obtaining top results across all datasets. The proposed method achieved about a 2% increase in accuracy almost across all categories compared to the GCPNet, and demonstrated a more than 5% improvement in quality compared to the UNet series. The major reason for this is the powerful encoding and decoding abilities provided by the integrated modules. The GCUNet's architecture incorporates the GR block, CG residual (CGR) block, and multi-path residual pooling, which allows for multi-scale dense feature extraction. The features from multiple levels are maximally retained, and the residual context attention module facilitates a high boundary intensity decoder for promising prediction map output. Thus, the GCUNet architecture consistently achieves the most advanced results overall.

**Figure 11:**
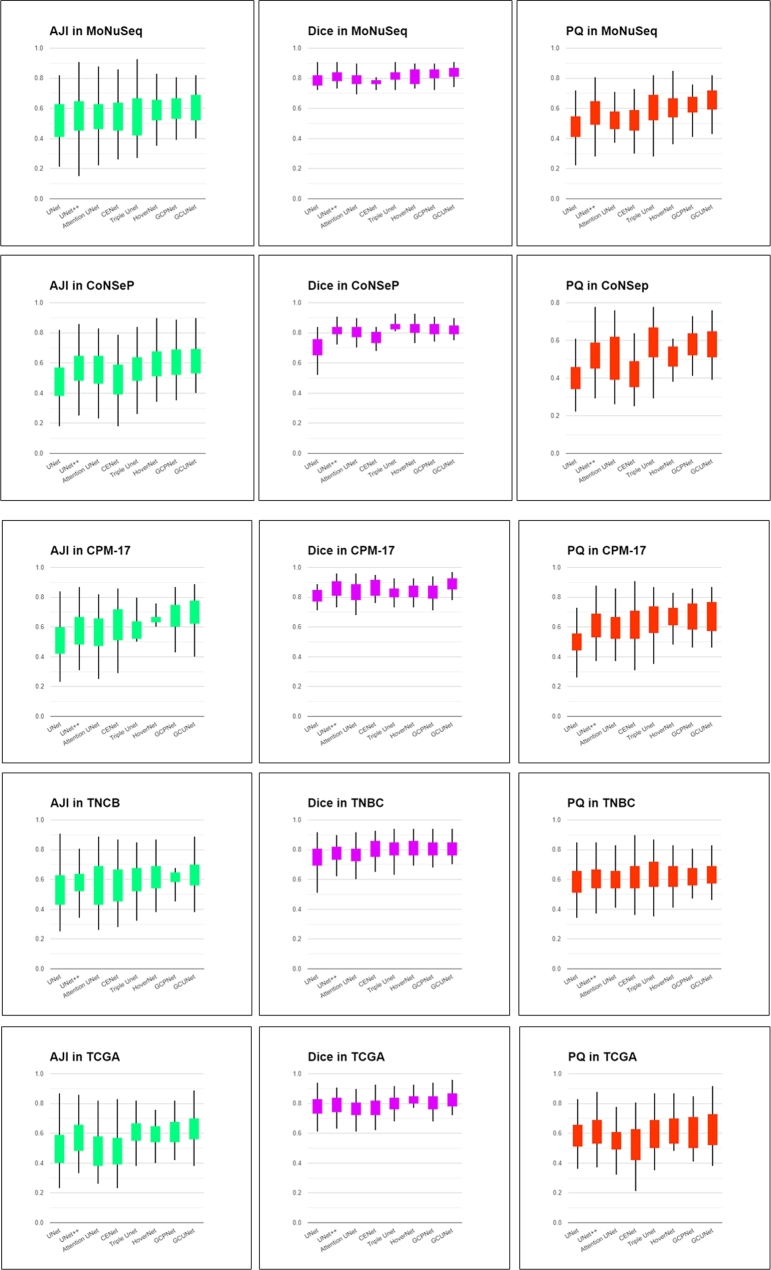
Boxplot image for a statistical distribution. The y means accuracy in (%) and the x means different methods.

**Figure 12:**
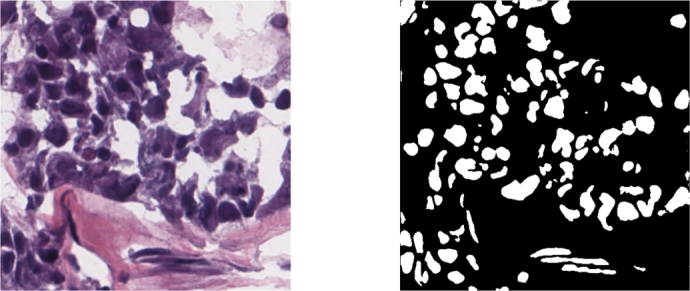
Visualization of proposed method in complicated situation. The left is inputting image, the right is the result label map.

A boxplot provides a statistical distribution of segmentation results. Fig. 11 presents the performance of segmentation results for each image in the test set across different models and datasets (ClusteredCell, MoNuSeg, CoNSeP, CPM-17, TCGA, and TNBC). Our method consistently outperforms others in each dataset, albeit with average variation in segmentation results for each image, further validating its superiority. Fig. 12 visualizes the results of proposed method.

### 4.7 Real diagnosis test

As we have demonstrated the superior performance of the proposed GC-UNet, we have deployed it in a real-world biomedical diagnosis setting. The results are showcased in Table 4. Given the absence of labeled data for this, we have utilized the precision rate to evaluate these results. The results illustrate a high rate of true positive outcomes (99.1%), a very limited number of false positives (0.5%), amounting to an impressive 99.5% precision rate. This suggests that the method can effectively be utilized in cervical nuclei segmentation, playing a crucial role in the diagnosis of cervical cancer. Moreover, the proposed method only takes 0.85 seconds per image, a processing speed that holds significant potential for practical, real-world application.

**Table 4:** Precision rate in real diagnosis.

	True Positive	False Positive	Precision Rate
GCUNet	99.1	0.5	99.5

## 5 Conclusion

The GCUNet demonstrated exceptional performance in accurate nuclei segmentation of biomedical images compared to other state-of-the-art methods. The strength of this work lies in the novel integration of the DenseNet backbone, CAGP block, CGR block, RCA module, and Multi-path Residual Pooling, all of which contribute to a significant enhancement in encoding ability and accurate nuclei segmentation. This research achieved 99.5% precision rate in real-world applications, further illustrating its potential in cervical cancer diagnosis.

While the results of our research are promising, it is important to acknowledge the limitations that present potential avenues for future research. A significant area to explore is instance-level segmentation. Although our method has successfully outlined the boundaries of cells, the instance information of each cell is missing. Future research can focus on creating a new dataset and accomplishing nuclei segmentation at the instance level. Therefore, while our GCUNet has made strides in improving nuclei segmentation accuracy, the journey towards a more comprehensive and precise solution continues.

## CRediT authorship contribution statement

Mr. Enguang Zhuang, Rixin Xie, Yuxin Bian, Jiayan Wang, Pengyi Tao and Heng Zhang performed the experiments, analyzed and interpreted the data; Dr. Shenlu Jiang conceived and designed the experiments; Contributed reagents, materials, analysis tools or data; and wrote the paper.

## Declaration of Competing Interest

The authors declare that they have no known competing financial interests or personal relationships that could have appeared to influence the work reported in this paper.

## Data Availability

No data was used for the research described in the article.
